# Colorimetric Detection of Sulfide Anions via Redox-Modulated Surface Chemistry and Morphology of Au-Hg Nanorods

**DOI:** 10.1155/2019/8961837

**Published:** 2019-05-02

**Authors:** Xiaotian Zhu, Chang Liu, Jie Liu

**Affiliations:** School of Materials Science and Engineering, Zhengzhou University, Zhengzhou 450001, China

## Abstract

A new colorimetric assay for the detection of sulfide anions with high sensitivity and selectivity is reported, utilizing Au-Hg alloy nanorods (Au-HgNRs) as probe. Au-HgNRs were prepared by modifying gold nanorods (AuNRs) with reducing agent and mercury ions. In an aqueous solution with sulfide anions, the formation of mercuric sulfide due to redox reaction between the amalgams and sulfide anions greatly changed the surface chemistry and morphology of the Au-HgNRs, leading to a red shift of the localized surface plasmon resonance (LSPR) absorption peak, accompanied by a change in colorimetric response. A good linear relationship was obtained between the LSPR peak wavelength shift and concentration of sulfide anion in the range of 1 × 10^−5^−1 × 10^−4 ^mol/L. The selectivity of this method has been investigated by other anions. The colorimetric sensing system successfully detected sulfide in wastewater from leather industry.

## 1. Introduction

Monitoring of inorganic anions such as S^2-^, BrO_3_^−^, ClO_2_^−^, ClO_3_^−^, Br^−^, and NO_2_^−^ in aquatic ecosystems is always an important issue because these anions exert adverse effects on the environment [1–3]. Among them, sulfide is one of the widespread toxic anion pollutants which comes from diverse sources including nature and human activities. The application of sulfur-containing chemicals in many industrial processes and immobilization of sulfur-containing minerals by bacteria are responsible for the presence of sulfide anions in wastewater effluents and natural waters, respectively [2]. Physiologically, sulfide anions pose a great threat to human health, like mucous membranes irritation, respiratory paralysis, and so on [3]. Increasing concerns over monitoring sulfide anions in aqueous solution have motivated the development of new methods with high selectivity, sensitivity, and rapidity. To date, several techniques are available for the detection of sulfide anion, such as chromatography [4, 5], electrophoresis [6], and fluorimetry [7]. These approaches are proved to be sensitive and selective. However, the development of these approaches usually requires sophisticated reactions and purification treatments, and their applications often rely on the use of expensive scientific instruments. In most cases, specific knowledge and skills are required for instrument operation and data assessment. Therefore, developing an accurate, fast-response, cost-effective sensor for on-site sulfide anion detection is helpful and still urgently needed.

Colorimetric methods are alternative simple approaches for measuring analytes in many applications because they require no costly and sophisticated equipment other than a spectrophotometer [8–13]. Recently, noble metal nanoparticles have attracted much attention in colorimetric detection because of their unique optical and physical properties. In particular, gold nanoparticles have rapidly become powerful platforms for the development of optical sensors because of their strong local surface plasmon resonance (SPR) and color-tunable behaviors. Several groups proposed colorimetric detection methods based on gold nanoparticles (AuNPs) for the assay of different kinds of analytes [12]. For instance, AuNPs have been shown to be versatile tools for colorimetric probing of Hg^2+^, Pb^2+^, Cu^2+^, and reducing agents [13–16], exhibiting high sensitivity and selectivity. The aggregation or surface modification of the gold nanomaterials upon interacting or reacting with analytes results in colorimetric responses caused by broadening, shifting, or intensity changing of the SPR peak.

More recently, on the basis of the surface modified gold nanoparticles, several sensors have proven to be effective for S^2-^ sensing. Zhang et al. exploited core/shell Cu@Au nanoparticles as sensors which can selectively recognize sulfide anion [8]. Wang et al. proposed a possible method of detection of S^2-^ based on gold nanorods (AuNRs) coated with mesoporous silica but failed to analyze S^2-^ in a quantitative manner [9]. In a similar way, Huang et al. achieved Au/Ag nanorods with core/shell structure, thereby realizing the detection of S^2-^ [10]. In these methods, the SPR wavelengths or intensities appreciably shift or change by slight change of the morphology, dispersity, or composition of the nanoparticles. However, some limitations still preclude the use of these methods in practical applications, such as complicated preparation procedures, time consuming analysis, and potential interferences. Moreover, very few studies have directly addressed the effects of structure and properties of the nanoparticles on sensing performance, though it is believed that this knowledge will help to develop more sensitive assays and to better understand the sensing mechanism [8–10].

In this work, based on the well-known amalgamation process that occurs between gold and mercury, Au-Hg nanorods (Au-HgNRs), alloy nanoparticles were prepared and stabilized with surfactant. Then we employed Au-HgNRs to detect S^2-^, resulting in a perceivable color change from cyan to green grey along with significant red shift of longitudinal SPR (LSPR) peak wavelength. The results indicate our new approach for the colorimetric detection of S^2-^ is fast, simple, quantitative, and selective. The practicality of this sensor for the detection of S^2-^ in wastewater from leather industry has also been validated.

## 2. Materials and Methods

### 2.1. Reagents

Hydrogen tetrachloroaurate (III) trihydrate (HAuCl_4_·3H_2_O, ≥99.9%), cetyltrimethylammonium bromide (CTAB, >98.0%), L-ascorbic acid (AA, >99%), 5-bromosalicylic acid (5-BrSA), and sodium sulfide hydrate (Na_2_S·9H_2_O) were purchased from Aladdin Biochemical Technology Co., Ltd. (Shanghai, China). Sodium borohydride (NaBH_4_, 98%) and silver nitrate (AgNO_3_, 99.999%) were obtained from Sinopharm Chemical Reagent (Shanghai, China). Mercury (II) chloride (HgCl_2_) was obtained from Tongren Yinhu Chemical Co., Ltd. (Guizhou, China). The other used salts NaCl, KI, Ba(C_2_O_4_)_2_, NH_4_F, and Na_2_SO_4_ were purchased from Kemiou Chemical Reagent Co., Ltd. (Tianjin, China). All chemicals used were of analytical reagent grade and used without further purification. All solutions were prepared with deionized water obtained from a Millipore Milli-Q water purification system.

### 2.2. Synthesis of AuNRs

AuNRs were synthesized according to the seed-mediated growth method reported in literature with slight modifications [17, 18]. In a typical synthesis process, the gold seeds were firstly prepared. Briefly, 10 mL of 0.5 mmol/L HAuCl_4_ solution was gently mixed with 10 mL of 0.2 M CTAB solution. Seed growth was initiated by rapidly adding 0.6 mL of ice cold 0.01 mol/L NaBH_4_ under continuous stirring, which led to a change in the color of solution from yellow to brownish-yellow. Two minutes later, the stirring was stopped. The seed solution was aged at room temperature for 2 h before use. Secondly, 9.0 g CTAB together with 0.9 g 5-BrSA was dissolved in 250 mL warm water (70°C) in a 1000 mL Erlenmeyer flask to prepare the growth solution. The solution was allowed to cool to 30°C, and then 12 mL of 4 mmol/L AgNO_3_ solution was introduced. Subsequently, 250 mL of 1 mmol/L HAuCl_4_ solution was added. After 15 min of slow stirring, 2 mL 0.064 mol/L AA was added, and the solution was vigorously stirred for 30 s until it became colorless. Finally, 0.8 mL of seed solution was added to growth solution. The resultant mixture was left undisturbed at 30°C for 12 h for nanorods growth. The resultant mixture was purified by centrifugation at 8000 rpm for 20 min to remove unreacted chemicals. The precipitates were redispersed in 10 mL of Milli-Q water.

### 2.3. Preparation of Au-HgNRs

Typically, 2 mL of the above prepared AuNRs solution (1.2 × 10^-3 ^mol/L) was mixed with 110 *μ*L HgCl_2_ solution, and then certain amount of 0.01 mol/L NaBH_4_ solution was added and the mixture was kept at 25°C for 24 h. To achieve complete reduction of Hg^2+^, excess amounts of reducing agent (NaBH_4_) have been used in all the experiments. The resultant mixture was centrifuged at 5000 rpm for 20 min to remove unreacted chemicals. The precipitates were redispersed in 10 mL of Milli-Q water and several drops of 0.1 M CTAB solution were added in order to stabilize the nanorods. Au-HgNRs prepared by adding 50, 100, and 150 *μ*mol/L Hg^2+^ were denoted as Au-HgNRs-I, Au-HgNRs-II, and Au-HgNRs-III, respectively. The Au-HgNRs solutions stored at room temperature showed long-term colloidal stability for several months (> 3 months).

### 2.4. Sulfide Anions Detection Test

The Au-HgNRs solution was suitably diluted with Milli-Q water before assaying, in order to achieve an absorbance of the LSPR band lower than 1.0. Afterwards, appropriate amount of sample solution was injected into the Au-HgNRs solution. After a quick mixing, the absorption spectra of the resulting solution were recorded on a UV-vis spectrometer (TU-1950, Purkinje General Instrument, Ltd., Beijing, China) using a 10-mm quartz cuvette in the wavelength range from 400 to 900 nm.

### 2.5. Transmission Electron Microscopy (TEM)

TEM analysis was conducted on a JEM-2100 electron microscope (JEOL, Tokyo, Japan) operating at an accelerating voltage 100 kV. The specimens were prepared by depositing drops of the nanorods solutions onto the surface of a carbon-coated copper grid and air-dried at room temperature. For statistical analysis of size, shape, and aspect ratio of the nanorods, at least 100 objects were counted for each sample.

## 3. Results and Discussion

### 3.1. Mechanisms for the Sensing System

**Figure 1:**
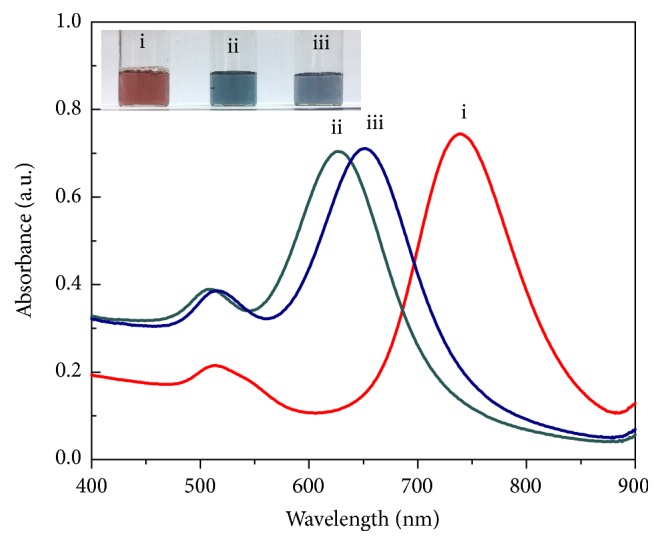
UV-vis absorption spectra of (i) CTAB-protected AuNRs, (ii) Au-HgNRs, and (iii) Au-HgNRs after addition of sulfide anions. The inset image shows the colorimetric response.

**Figure 2:**
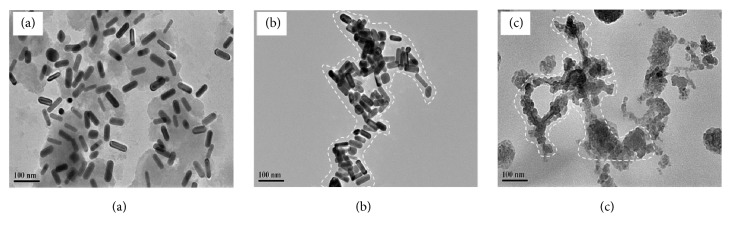
TEM images of (a) AuNRs, (b) Au-HgNRs, and (c) Au-HgNRs in the presence of S^2-^.

The CTAB-protected AuNRs are positively charged and stable in aqueous solution. It has been reported that CTAB could even protect the AuNRs from aggregation in a solution with relatively high concentration of salt [19]. When NaBH_4_ was introduced into AuNRs/Hg^2+^ mixture, the reduction of Hg^2+^ to Hg^0^ occurred, whereafter the strong affinity between Au^0^ and Hg^0^ alters the surface chemistry as well as the aspect ratio of AuNRs, leading to a remarkable blue shift in the LSPR peak of AuNRs and changes in solution color [20]. It can be clearly seen from Figure 1 that the inputs of Hg^2+^ and NaBH_4_ induced both an obvious blue shift (~140nm) in the LSPR peak of AuNRs and a red-to-blue color change (see inset in Figure 1), indicating that an amalgamation between Au and Hg may take place. Experimental evidence of the amalgamate formation was further obtained via TEM analysis (Figure 2); the presence of Hg^2+^ and reducing agent decreases the nanorods' length and reduces their aspect ratio (length/diameter) from 2.69 to 1.67. This behavior has been successfully exploited to detect trace amount of Hg^2+^ in water and in biological systems [20]. Lin and coworkers [21] used gold nanoparticles (AuNPs) to detect Hg^2+^, based on the reduction of Hg^2+^ by mild reducing agent to form Hg-Au amalgam-like structure on the surface of the AuNPs. Jin and Bi et al. [13, 22] have studied the optical detection mechanism of Hg^2+^, using AuNRs as probes. Such a method allowed one to achieve a LOD for Hg^2+^ at ppt level. These findings suggest that surface chemistry modification of AuNPs or AuNRs could be a promising strategy for colorimetric detection applications.

On adding S^2-^ to the solution of Au-HgNRs, significant red shifts in the coupling plasmon wavelength were observed from Figure 1. Besides, the color of the solution varied appreciably from cyan to green grey. The spectra and color changes may be attributed to the strong binding affinity of sulfide towards the surface of the Au-HgNRs. The TEM image of Au-HgNRs (Figure 2) shows that the nanorods suffered significant morphology transformations after the addition of S^2-^, leading to an aggregation of nanorods covered with a thick shell. In Figure 2(c), the rod-like structure of Au-HgNRs is almost unperceivable in the TEM image. This can be explained by the fact that the distribution of compounds formed on the surface of Au-HgNRs was not uniform. Nevertheless, the strong transversal SPR (TSPR) and LSPR absorption spectrum acquired from the Au-HgNRs after the addition of sulfide indicates the presence of anisotropic nanorods-like structure in the final product. In this manner, sulfide can be detected via monitoring the LSPR absorption and color change of the alloy nanorods even by the naked eye.

**Scheme 1:**
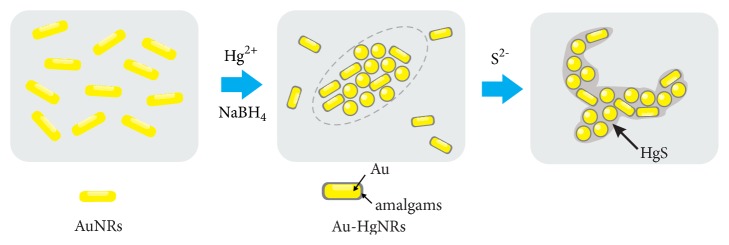
Illustration of colorimetric sensing mechanism based on the redox reaction modulated surface chemistry and morphology of Au-HgNRs nanorods.

The overall possible sensing mechanism for the detection of S^2-^ is depicted in Scheme 1. The variation of the detectable colorimetric signal mainly derives from the redox-induced changes in chemistry and morphology of the alloy nanorods. In the aqueous solution, the total dissolved sulfide is present as the unionized form (H_2_S) and as ionized forms (HS^−^ and S^2-^). Considering the pH changes of the sulfide solution upon mixing with CTAB-protected Au-HgNRs, the possible chemical reactions involved in the observed phenomenon can be expressed as follows:

(1) $$\begin{array}{c} Au\text{-}Hg+S^{2-}+\frac{1}{2}O_{2}+2H^{+}\to \\ Au\text{-}HgS+H_{2}O\\ Au\text{-}Hg+{HS}^{-}+\frac{1}{2}O_{2}+H^{+}\to \\ Au\text{-}HgS+H_{2}O\\ Au\text{-}Hg+H_{2}S+\frac{1}{2}O_{2}\to \\ Au\text{-}HgS+H_{2}O \end{array}$$

Based on this assumption, the presence of H_2_S and HS^−^ due to the equilibrium for H_2_S/HS^−^/S^2-^ in aqueous solution as a function of pH does not affect the redox reaction on the Au-HgNRs surface. For the noble metal nanorods, the SPR is highly sensitive to their dielectric environment and therefore potentially suitable for sensor applications [23–25]. When the sulfide solution was added, certain part of mercury in amalgams was oxidized to HgS. The formation of HgS on the surface of Au-HgNRs consequently caused a remarkable change in the effective dielectric constant of the alloy nanorods, leading to a shift of the LSPR band towards longer wavelengths. Meanwhile, the TSPR of Au-HgNRs red-shifted from 505 nm to 515 nm for the same reason. Jakse et al. reported that the reaction between mercury and sodium sulfide could induce rapid HgS formation [26]. Such a feature can be compatible with the quick response of spectra upon the addition of S^2-^ into the Au-HgNRs solutions.

### 3.2. Effect of Au-HgNRs on Sensing Performance

**Figure 3:**
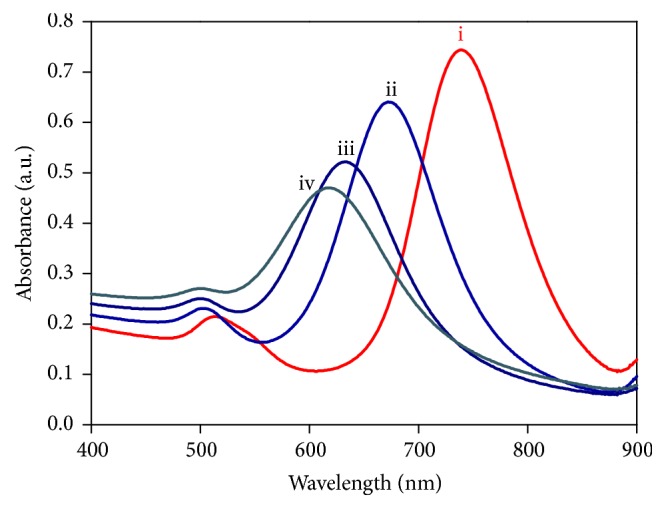
UV-vis absorption spectra of (i) as-prepared AuNRs, (ii) Au-HgNRs-I, (iii) Au-HgNRs-II, and (iv) Au-HgNRs-III.

Colloidal stability is always a prerequisite when considering potential applications of nanoparticle dispersions as sensing probes [27]. The AuNRs prepared via a seed-mediated sequential growth process are capped by a double layer of CTAB, and this layer provides a short-range electrostatic repulsive force to prevent the strong aggregation of AuNRs in solution. Though the dynamic CTAB layer is considered to be not thermodynamically stable in water [28], acceptable colloidal stability was still found by maintaining suitable amount of CTAB in Au-HgNRs solutions. Upon the addition of Hg^2+^ and NaBH_4_, the LSPR peak of AuNRs blue-shifted gradually, accompanied by a decrease of the peak. It was further observed that an increase in the concentration of Hg^2+^ led to more distinctive blue shifts. The characteristic LSPR peaks of the as-prepared AuNRs, Au-HgNRs-I, Au-HgNRs-II, and Au-HgNRs-III locate at 740 nm, 680 nm, 640 nm, and 620 nm, respectively (Figure 3). Most of the previous studies attribute this phenomenon to preferential absorption of CTAB on the lateral sides of the nanorods, and therefore the amalgamation between Au^0^ and Hg^0^ will preferentially occur at the tips of the nanorods [13, 20].

**Figure 4:**
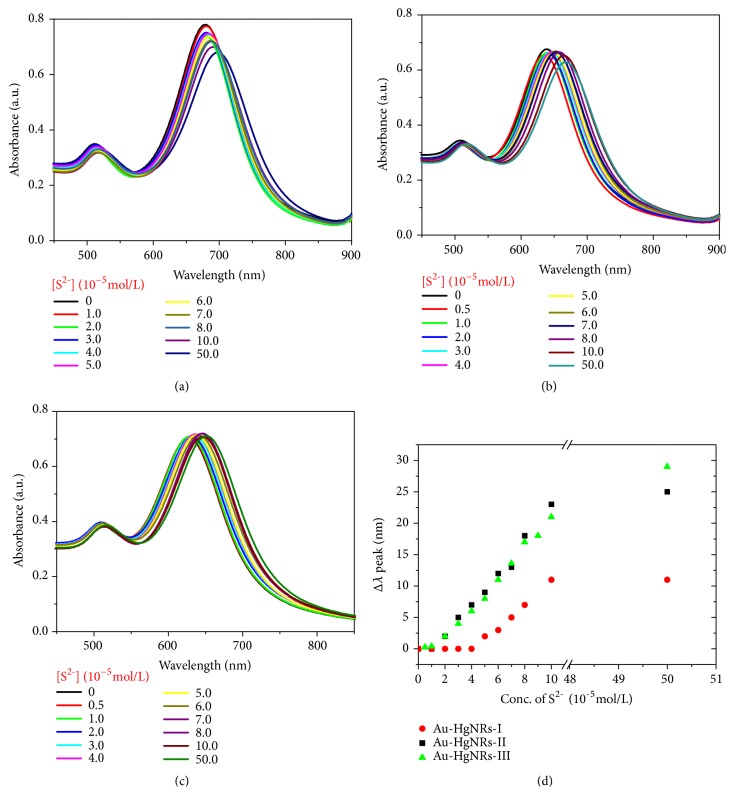
UV-vis absorption spectra of (a) Au-HgNRs-I, (b) Au-HgNRs-II, and (c) Au-HgNRs-III solutions containing different concentrations of S^2-^; (d) variation plots of shifts in LSPR peak wavelength (Δ*λ*) against the concentration of S^2-^.

Figure 4 shows the absorption spectra of the Au-HgNRs solutions after 2 min of mixing with S^2-^. Monitoring of the absorption spectra of Au-HgNRs over a period of 30 min showed no further wavelength shifts. It was observed that the red shifts in the LSPR peak are significantly different for the alloy nanorods with different initial LSPR peaks. When the Au-HgNRs have longer LSPR peak wavelength, as shown in Figure 4(a), the LSPR peak of the Au-HgNRs-I nanorods exhibited a limited red shift from 680 nm to 690 nm. In Figures 4(b) and 4(c), compared with the first measurement in Figure 4(a), there are significantly red shifts in the absorption spectra by using Au-HgNRs with shorter LSPR peak wavelength.  Figure 4(d) presents the relationship between the shift of the LSPR peak wavelength (Δ*λ*) and the concentration of S^2-^. The simulated calibration plots displayed good linear relationship between Δ*λ* and the concentration of S^2-^ in different ranges. The increase of Δ*λ* in sensing process with the increase of Hg^2+^ used in the preparation of Au-HgNRs indicates that more HgS may be deposited on the surface of the nanorods. Though the mechanisms of the colorimetric responses all involving the redox reaction modulated surface chemistry of the alloy nanorods, it was found that the shortening of LSPR peak wavelength amplifies the change of plasmon signal due to the presence of more amalgams on the surface of nanorods, consequently offering higher sensitivity as well as broader detection range.

### 3.3. Optimization of Conditions for S^2−^ Sensing

**Figure 5:**
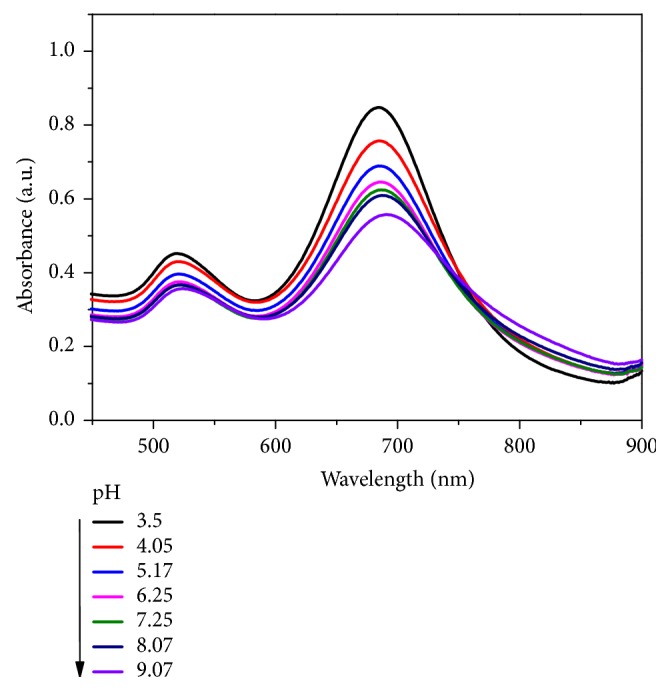
UV-vis absorption spectra of Au-HgNRs upon the addition of S^2-^ under different pH.

The pH of as-prepared Au-HgNRs-III solution with the addition of CTAB was determined to be 3.50. The effect of pH on the spectra changes of CTAB-protected Au-HgNRs in the absence and presence of S^2-^ (5 × 10^-4 ^mol/L) was investigated. The pH of the system was adjusted by using NaOH solution. As shown in Figure 5, with the increase of pH from 3.50 to 9.07, the absorption values at LSPR peak gradually decreased. It can also be seen from the spectra that an increase in pH leads to slight red shifts in both the TSPR and LSPR absorption during the detection period. This finding was possibly due to the presence of CTAB in the solution. In the preparation of AuNRs, CTAB acts as structure-directing and stabilizing agent by forming a bilayer on the surface of nanorods [29, 30]. The removal of excess free CTAB in aqueous solution by centrifugation has been shown to result in insufficient stability of the nanorods against aggregation, because the bound CTAB layer will gradually enter the solution [26, 27, 31]. Though in some cases CTAB needs to be removed or replaced from the surface of nanorods for many applications owing to its cytotoxicity [32], we still prefer to use CTAB to keep the nanorods solution stable. Since Au-HgNRs are stabilized by micelles of CTAB and CTAB cations gathering around the nanorods, the introduction of NaOH into the positively charged nanorods leads to an adsorption of OH^−^ onto the surface of nanorods as counterions around the surface. Hence, the electrostatic attraction between the OH^−^ anions and CTAB cations on the surface of nanorods may cause neighboring nanorods to aggregate. Huang et al. observed a similar red shift in the plasmon band along with the decrease in the absorption values by forming an end-to-end Na_3_PO_4_-induced gold nanorods assembly [19]. Therefore, pH of 3.50 was finally selected in the following measurements.

**Figure 6:**
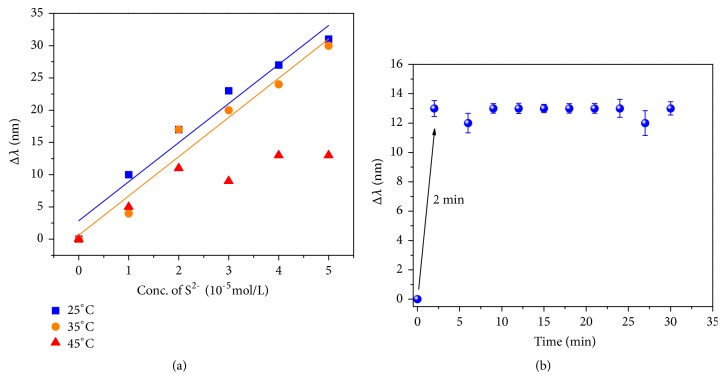
The effect of (a) detection temperature and (b) time on Δ*λ* of the Au-HgNRs sensing system for S^2-^.

The temperature effect on the sensing system was also explored over a temperature range from 25 to 45°C due to the possible reshaping phenomena of the metal nanorods under thermal treatment [33]. When the temperature increased in the tested range, the decrease in the Δ*λ* of the system was observed (Figure 6(a)). At 45°C, the significant deviation from linearity in the relationship between Δ*λ* and concentration of S^2-^ appears to be a direct consequence of the influence of temperature on the morphology, composition, and state of dispersion of the nanoparticles [34]. The release of hydrogen sulfide with the increase of temperature may also be responsible for the decreased sensing performance for sulfide anions. At a fixed temperature of 25°C, Δ*λ* increased with the increasing of reaction time and reached the maximum at only 2 min as shown in Figure 6(b). The fast-response characteristics of the sensing system demonstrated that the redox reaction between amalgams and sulfide is very fast at room temperature. Taking these factors into account, the optimal detection conditions for the assay of sulfide anions in solution were chosen to be at 25°C and 2 min incubation.

### 3.4. Selectivity and Sensitivity for Determination of Sulfide Anions

**Figure 7:**
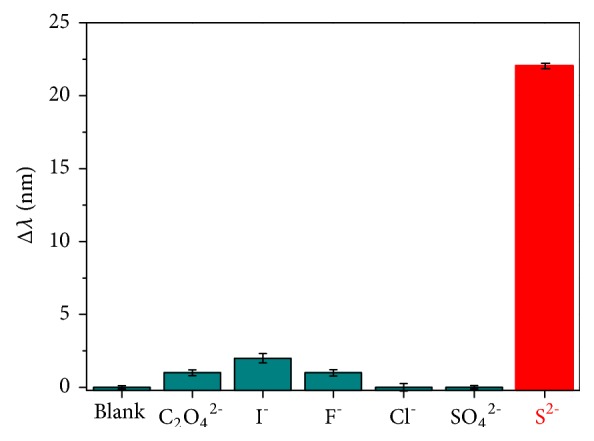
Selectivity of the Au-HgNRs sensing system for S^2-^ over several anions. The concentration of each anion was 5 × 10^−4 ^mol/L.

To realize the selectivity of the sensing system based on redox-modulated surface chemistry and morphology of the CTAB-protected Au-HgNRs towards sulfide anions, we examined different common anions under optimal detection conditions.  Figure 7 shows Δ*λ* of Au-HgNRs on adding S^2-^, I^−^, SO_4_^2−^, F^−^, C_2_O_4_^2−^, and Cl^−^ ions. Only S^2-^ caused the LSPR peak wavelength of solution to significantly red shift, indicating that Au-HgNRs is selective for S^2-^. As shown in Figure 7, the addition of 5 × 10^-4 ^M S^2-^ to the Au-HgNRs solution resulted in a value of Δ*λ* as 22 nm, whereas the other anions tested (5 × 10^-4 ^mol/L) gave values similar to the blank, indicating that other anions at the same concentration did not interfere with the determination of sulfide anions.

**Figure 8:**
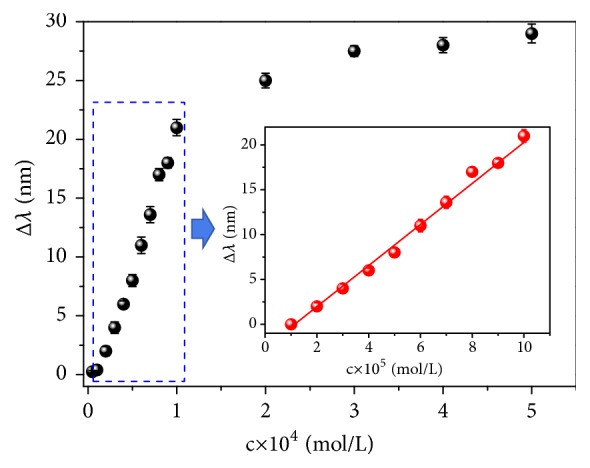
The relationship between Δ*λ* and the concentration of S^2-^. Inset: standard curve of detection of sulfide based on Au-HgNRs sensing system.

Under the optimum detection conditions, we detected a series of standard sulfide stock solutions at concentrations from 1 × 10^−5^ to 5 × 10^-4 ^mol/L (Figure 8). As the concentration of sulfide anions increases, the Δ*λ* value of the solution first increases rapidly and then plateaued. The inset in Figure 8 shows the linear relationship between Δ*λ* and the concentration of S^2-^ over the range of 1 × 10^−5^~ 1 × 10^-4 ^mol/L. The linear equation was Δ*λ*=2.67 × 10^5^c−3.53; the resulting correlation coefficient R^2^ was 0.993. The limit of detection (LOD) was calculated based on the 3*σ* method. The LOD for sulfide anions was calculated to be 2.5 × 10^−6^ mol/L.

### 3.5. Application in Wastewater from Leather Industry

**Figure 9:**
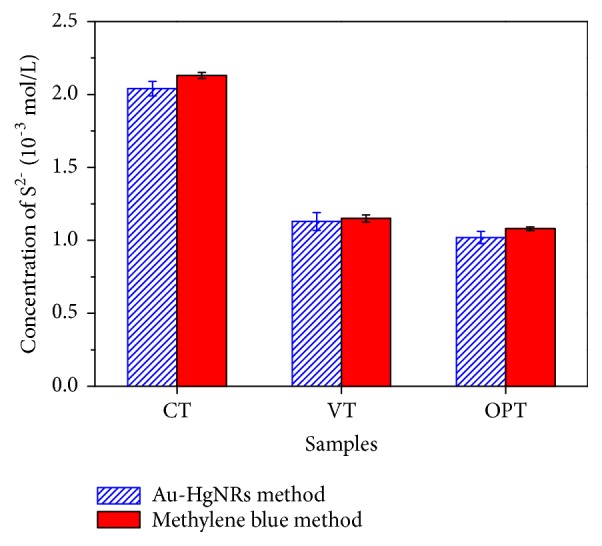
Determination of concentrations of S^2-^ in real samples (CT: wastewater from chrome tanning process; VT: wastewater from vegetable tanning process; OPT: wastewater from organic phosphine tanning process).

To evaluate the potential of Au-HgNRs for sulfide anions detection in real samples, one possible application in tannery wastewater analysis was attempted. In leather industry, sodium sulfide is the most commonly used and effective reagent to eliminate hair or other components that are not transformed into leather [35]. Wastewater samples were collected from chrome, vegetable, and organic phosphine tanning processes from different factories in Jiaozuo City. Before the samples were used, they were filtered through a Micropore filter (0.22 *μ*m) and suitably diluted with Milli-Q water. As shown in Figure 9, under the optimal experimental conditions, the results were basically consistent with the methylene blue method (GB/T16489-1996) results, thus showing the applicability of the Au-HgNRs for determination of S^2-^ concentrations in wastewater.

## 4. Conclusions

A simple, fast, and selective method of detecting S^2-^ was developed based on the redox-modulated surface chemistry and morphology of Au-HgNRs. The presence of S^2-^ can be monitored by colorimetric response of the Au-HgNRs. This method shows relatively good selectivity for S^2-^ over other common anions with a LOD of 2.5 × 10^−6^ mol/L. Under the optimal experimental conditions, this assay can be used for detection of S^2-^ in wastewater from leather industry, indicating that the proposed Au-HgNRs based method has the potential to be applied for wastewater quality monitoring.

## Data Availability

The data used to support the findings of this study are included within the article.
